# Identification of functional domains of the minor fimbrial antigen involved in the interaction of *Porphyromonas gingivalis* with oral streptococci

**DOI:** 10.1111/omi.12280

**Published:** 2020-02-13

**Authors:** Mohammad Roky, John O. Trent, Donald R. Demuth

**Affiliations:** ^1^ Department of Oral Immunology and Infectious Diseases University of Louisville School of Dentistry Louisville KY USA; ^2^ Department of Microbiology and Immunology University of Louisville School of Medicine Louisville KY USA; ^3^ Department of Medicine University of Louisville School of Medicine Louisville KY USA

**Keywords:** adherence, biofilm, Mfa1, *P. gingivalis*, streptococci

## Abstract

*Porphyromonas gingivalis* is associated with chronic periodontitis and may initially colonize the oral cavity by adhering to streptococci. Adhesion to streptococci is driven by interaction of the minor fimbrial antigen (Mfa1) with streptococcal antigen I/II. We identified the region of antigen I/II required for this interaction and developed small molecule mimetics that inhibited *P. gingivalis* adherence. However, the functional motifs of Mfa1 involved in the interaction with antigen I/II remain uncharacterized. A series of N‐ and C‐terminal peptide fragments of Mfa1 were expressed and tested for inhibition of *P. gingivalis* adherence to *S. gordonii*. This approach identified residues 225–400 of Mfa1 as essential for *P. gingivalis* adherence. Using the three‐dimensional structure of Mfa1, a putative binding cleft was identified using SiteMap and five small molecule mimetics could dock in this site. Site‐specific mutation of residues in the predicted cleft, including R240A, W275A, D321A and A357P inhibited the interaction of Mfa1 with streptococci, whereas mutation of residues not in the predicted cleft (V238A, I252F and ΔK253) had no effect. Complementation of an Mfa1‐deficient *P. gingivalis* strain with wild‐type *mfa1* restored adherence to streptococci, whereas complementation with full‐length *mfa1* containing the R240A or A357P mutations did not restore adherence. The mutations did not affect polymerization of Mfa1, suggesting that the complemented strains produced intact minor fimbriae. These results identified specific residues and structural motifs required for the Mfa1‐antigen I/II interaction and will facilitate the design of small molecule therapeutics to prevent *P. gingivalis* colonization of the oral cavity.

## Graphical abstract

*Porphyromonas gingivalis* adheres to oral streptococci through the interaction of Mfa1 with the BAR domain of streptococcal Ag I/II. In silico analysis of the Mfa1 crystal structure and peptide mapping of the full length protein identified a putative ligand binding cleft in the central region of Mfa1 comprising residues 226–400. Five different small molecule mimetics of the BAR domain that function as potent inhibitors of *P. gingivalis* adherence could be docked at this site. The functional properties of the site were confirmed by site specific mutagenesis and several amino acids required for *P. gingivalis* adherence were identified. Complementation of a Mfa1‐deficient *P. gingivalis* strain with full length wild type *mfa1* restored *P. gingivalis* adherence whereas strains complemented with the site specific *mfa1* mutants did not. Our results define a domain of Mfa1 that mediates interaction with AgI/II and is essential for *P. gingivalis* adherence to streptococci.

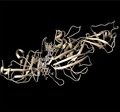

## 1 INTRODUCTION

Periodontal disease is the sixth most prevalent disease in the world and approximately 50% of adults in the United States suffer from some form of periodontitis (Eke, Dye, Wei, Thornton‐Evans, & Genco, 2012; Kassebaum et al., 2014). The human oral cavity is home to ~700 species of bacteria and maintaining host/microbe homeostasis is key to maintaining periodontal health. *Porphyromonas gingivalis* is strongly associated with chronic adult periodontitis and is an important pathogen that is capable of modulating the host immune response and disrupting normal host/microbe homeostasis (Hajishengallis, 2015; Olsen, Lambris, & Hajishengallis, 2017). This can lead to the development of a dysbiotic microbial community which can induce uncontrolled inflammation leading to the destruction of tooth supporting tissues, and ultimately tooth loss (Hajishengallis & Lamont, 2014, 2016; Lamont & Hajishengallis, 2015). Periodontitis is also associated with increased risk of other systemic diseases such as rheumatoid arthritis, cardiovascular disease, some cancers and chronic respiratory disease (Bingham & Moni, 2013; Kim & Amar, 2006; Winning & Linden, 2017).

The primary niche for *P. gingivalis* is the subgingival pocket but the organism also adheres efficiently to supragingival bacteria such as various commensal streptococci (Brooks, Demuth, Gil, & Lamont, 1997; Demuth, Irvine, Costerton, Cook, & Lamont, 2001; Lamont, Hersey, & Rosan, 1992). Indeed, adherence to streptococci can modulate the pathogenic potential of *P. gingivalis* (Daep, Novak, Lamont, & Demuth, 2011; Kuboniwa et al., 2017; Kuboniwa & Lamont, 2010) and may also be important for the initial colonization of the oral cavity by the organism. Initial colonization of the oral cavity by *P. gingivalis* is thought to occur at more available sites such as the supragingival tooth surface (Quirynen et al., 2005; Socransky, Haffajee, Ximenez‐Fyvie, Feres, & Mager, 1999; Takazoe, Nakamura, & Okuda, 1984) and oral introduction of *P. gingivalis* in human volunteers results in the organism locating almost exclusively on streptococcal‐rich supragingival plaque (Slots & Gibbons, 1978). In addition, in patients with periodontal disease, the levels of supragingival *P. gingivalis* have been shown to correlate with subgingival levels of the organism (Mayanagi, Sato, Shimauchi, & Takahashi, 2004). Thus, adherence of *P. gingivalis* to streptococci represents a viable target for therapeutic intervention.

*P. gingivalis* adherence to streptococci is driven by a protein–protein interaction between the minor fimbrial antigen, Mfa1, and the streptococcal antigen I/II protein (Brooks et al., 1997; Chung, Demuth, & Lamont, 2000; Demuth et al., 2001; Park et al., 2005). Deap et al. identified several discrete structural motifs in SspB that are essential for adherence and suggested that this functional region resembles the eukaryotic nuclear receptor (NR) box protein–protein interaction domain (Daep, Lamont, & Demuth, 2008). In addition, a synthetic peptide (BAR) that encompasses this region potently inhibited *P. gingivalis*/streptococcal adherence in vitro and significantly reduces *P. gingivalis* virulence in vivo (Daep et al., 2011). Subsequently, small molecule BAR peptidomimetics that potently inhibit *P. gingivalis* adherence were developed (Patil, Luzzio, & Demuth, 2015; Patil, Tan, Demuth, & Luzzio, 2016). Although the binding region in antigen I/II has been well characterized, little is known about the binding domains or motifs of Mfa1 that contribute to this protein–protein interaction.

In this study, N‐ and C‐terminal truncated Mfa1 polypeptides were shown to inhibit *P. gingivalis*/streptococcal adherence and suggested that Mfa1 functional motifs are present between residues 225–400 of the protein. Using the three‐dimensional structure of Mfa1 (Hall, Hasegawa, Yoshimura, & Persson, 2018), a putative binding cleft was identified using the prediction tool SiteMap. Site‐specific mutation of amino acids in the predicted cleft, for example, R240A, W275A, D321A and A357P inhibited the interaction of Mfa1 with streptococci. Finally, complementation of an Mfa1‐deficient *P. gingivalis* strain with wild‐type *mfa1* restored adherence to streptococci, whereas complementation with the site‐specific *mfa1* mutants resulted in significantly reduced levels of adherence. Together, these results identify specific residues and motifs that are important for the Mfa1/SspB protein–protein interaction.

## 2 METHODS

### 2.1 Bacterial strains and growth conditions

The strains and plasmids used in this study are shown in Table 1. *P. gingivalis* ATCC 33277 was grown in TSBY medium comprised of trypticase soy broth (Difco) supplemented with 2% yeast extract, 1 µg/ml hemin and 5 µg/ml menadione. For growth on plates, this medium was further supplemented with 1.5% agar and 5% sheep blood. The Mfa1‐deficient and complemented strains were cultured in medium containing the appropriate antibiotics, that is, 1 and 5 µg/ml of tetracycline and erythromycin, respectively. All *P. gingivalis* cultures were incubated under anaerobic conditions (10% CO_2_, 10% H_2_ and 80% N_2_). Brain heart infusion agar (Difco) supplemented with 5% yeast extract was used to grow S*. gordonii* DL‐1. *E. coli* strains were maintained in LB medium supplemented with the appropriate antibiotic. Where necessary, the final concentration of ampicillin was 100 µg/ml. All bacterial stocks were stored at −80°C in the appropriate medium supplemented with 30% glycerol.

**Table 1:** Strains and plasmids used in this study

	Strain or Plasmid	Characteristic	Source reference
*S. gordonii* DL1			Lab stock (Pakula & Walczak, 1963)
*P. gingivalis*	33277	ATCC	
	SMF1	Derivative of 33277 with insertional inactivation of the *mfa1* gene; Emr	(Lamont et al., 2002)
	cSMF1	SMF1 containing pTCOW‐Mfa1, complemented strain; Emr Tcr	(Park et al., 2005)
	cSMF1‐R240A	SMF1 containing pTCOW‐Mfa1 with the R240A mutation, complemented strain; Emr Tcr	This study
	cSMF1‐A357P	SMF1 containing pTCOW‐Mfa1 with mutation the A357P mutation, complemented strain; Emr Tcr	This study
*E coli*	XL1 blue cells		Agilent
	BL21 (DE3) pLysS		
Plasmid	pGEX6p1	GST tag expression vector	
	pG‐Mfa1	pGEX6p1 containing mfa1 (residues 21–563)	This study
	pG‐N194	pGEX6p1 containing N‐terminal mfa1 fragment, residues 21–194	This study
	pG‐N225	pGEX6p1 containing N‐terminal mfa1 fragment, residues 21–225	This study
	pG‐N279	pGEX6p1 containing N‐terminal mfa1 fragment, residues 21–279	This study
	pG‐N400	pGEX6p1 containing N‐terminal mfa1 fragment, residues 21–400	This study
	pG‐C280	pGEX6p1 containing C‐terminal mfa1 fragment, residues 280–563	This study
	pT‐COW	Shuttle vector plasmid; Amp^R^ Tc^R^ in *E. coli*; Tc^R^ in *P. gingivalis* Mob+Rep+	(Gardner, Russell, Wilson, Wang, & Shoemaker, 1996)
	pTCOW‐Mfa1	pT‐COW containing a 2.5‐kb fragment containing the upstream and coding region of the *mfa1* gene	(Park et al., 2005)
	pTCOW‐Mfa1 Arg240/Ala	pT‐COW‐Mfa1 containing the R240A mutation	This study
	pTCOW‐Mfa1 Ala357/Pro	pT‐COW‐Mfa1 containing the A357P mutation	This study

### 2.2 Recombinant protein/peptide constructs

Nucleotide primers used in this study are shown in Table 2. To generate the full‐length Mfa1 construct lacking the signal sequence, the *mfa1* sequence from 61 bp to 1689 bp was amplified using the forward and reverse primers listed in Table 2 from *P. gingivalis* ATCC33277 genomic DNA using platinum PCR supermix (Invitrogen) according to the manufacturer's instructions. Briefly, initial denaturation was carried out at 94°C for 3 min. This was followed by 30 cycles comprised of denaturation at 94°C for 30 s, primer annealing at 55°C for 30 s and strand extension at 72°C for 90 s. The amplified products were subsequently digested with *Sal*I and *Xho*I, ligated into the pGEX6p‐1 expression vector and transformed into chemically component *E. coli* BL21. For the construction of the Mfa1 N‐terminal and C‐terminal peptide fragments N194, N225, N279, N400 and C280, *mfa1* gene fragments encoding residues 21–194, 21–225, 21–279, 21–400 and a C‐terminal fragment encoding residues 280–563 of Mfa1 were amplified using the primers shown in Table 2 from *P. gingivalis* ATCC33277 genomic DNA. A similar approach to that described above was followed to clone these fragments in pGEX6p1 expression vector and introduce the constructs into *E. coli* BL21.

**Table 2:** Primers used in this study. Restriction enzyme sites are shown in italics

Protein/peptide name	Primer sequence
Mfa1	FP: 5′ ATTA *GTCGAC* AGTAAAGAGGGCAATGGCCCCGATCCG 3′
	RP: 5′ ATGG *CTCGAG* TAA GAGATCAACCTCATAG 3′
N194	FP: 5′ ATTA *GTCGAC* AGTAAAGAGGGCAATGGCCCCGATCCG 3′
	RP: 5′ GAAT *CTCGAG* TAA ACCATTCTTTTTGGCAATC 3′
N225	FP: 5′ ATTA *GTCGAC* AGTAAAGAGGGCAATGGCCCCGATCCG 3′
	RP: 5′ TAAT *CTC GAG* TAA CCCTGCGATAGCATTGGCCTCGGATA 3′
N279	FP: 5′ ATTA *GTCGAC* AGTAAAGAGGGCAATGGCCCCGATCCG 3′
	RP: 5′ ACCT *CTCGAG* TAA TTGAGCAACAACCCATCTGA 3′
N400	FP: 5′ ATTA *GTCGAC* AGTAAAGAGGGCAATGGCCCCGATCCG 3′
	RP: 5′ ATTA*CTCGAG*TTATTCTTTCTTGGGAGTAAACTTCGCACGAACC 3′
C280	FP: 5′ AAAA *GTCGAC* GGAGAACGTCGCCAATACCT 3′
	RP: 5′ ATGG *CTCGAG* TAA GAGATCAACCTCATAG 3′
N279‐R240A	FP: 5′‐CGT TCT GTA GCA **GCT** GCG ATG GTT TCA−3′
	RP: 5′‐CGT TGA AAC CAT CGC **AGC** TGC TAC AGA ACG CTC−3′
N279‐W275A	FP: 5′‐ATT ACG GAT ATC AGA **GCG** GTT GCT CAA GGA−3′
	RP: 5′‐TCC TTG AGC AAC **CGC** TCT GAT ATC CGT AAT−3′
N400‐D321A	FP: 5′ GCT ACC GAG TAT **GCT** TAT GCC GGT CTG TGG 3′
	RP: 5′ CAG ACC GGC ATA **AGC** ATA CTC GGT AGC ATT TGT 3′
N400‐A357P	FP: 5′ ACT GGC GAA TTG **CCA** AAT GCT CTT TCA 3′
	RP: 5′ TGA AAG AGC ATT **TGG** CAA TTC GCC AGT CAC 3′

### 2.3 Expression and purification of truncated Mfa1 peptides

To express the full‐length and truncated Mfa1 proteins, an overnight culture containing the desired construct was diluted in pre‐warmed LB medium supplemented with 100 µg/ml ampicillin to an OD_600 nm_ of 0.1 and incubated at 37°C in a rotating shaker at speed of 220 rpm. When the OD_600 nm_ reached 0.5, protein expression was induced by adding IPTG to a final concentration of 1mM. After further incubation for 4 hr at 37°C, cells were harvested by centrifugation at 3,000 *g* for 10 min and the cell pellets were frozen. One gram of frozen cell pellet was suspended in 5 ml of CellLytic B (Sigma‐Aldrich) containing lysozyme (0.2 mg/ml) and Benzonase (50 U/ml). A protease inhibitor cocktail (Thermo Fisher Scientific) was added as per manufacturer recommendations and incubated at 25°C for 30 min with gentle shaking. To complete the disruption of the cells, brief sonication was carried out using a Vibra‐Cell ultrasonic Liquid Processor VCX 130 (Sonics). Cells were pulsed at 20 kHz for 2 min using a 10 s short burst followed by a 30 s cooling interval. During sonication, all steps were carried out in ice. Cell debris was removed by centrifugation at 13,000 *g* for 20 min and the supernatant was transferred to a fresh tube.

Purification of GST‐tagged fusion proteins was carried out using the Pierce GST Spin Purification Kit (ThermoFisher). Briefly, spin columns were equilibrated with the equilibration/wash buffer supplied with the kit at 4°C, and then 15 ml of cell lysate supernatant was added to each column and incubated overnight at 4°C. Columns were washed three times with 15 ml of wash buffer by centrifugation at 700 *g* for 2 min. In column, cleavage with Precision Protease (ThermoFisher) was carried out by overnight incubation at 4°C to generate the truncated Mfa1 proteins. The Mfa1 polypeptides were eluted by centrifugation at 700 *g* for 2 min. The eluted peptides were subsequently chromatographed in disposable PD‐10 Desalting Columns (GE Healthcare) to remove salts. Purity of the eluted proteins was assessed using SDS‐PAGE electrophoresis and quantification of proteins was carried out using the BCA assay (Pierce).

### 2.4 Dual species biofilm model

Interspecies adherence and biofilm formation between *P. gingivalis* and *S. gordonii* were carried out essentially as previously described (Patil et al., 2016). *S. gordonii* cells were harvested from a 10 ml of overnight culture by centrifugation at 3,000 *g* for 5 min, washed with 10 ml of pre‐reduced PBS (10 mM Na_2_HPO_4_, 18 mM KH_2_PO_4_, 1.37 M NaCl and 2.7 mM KCl, pH 7.2) and suspended in 1 ml of PBS. Suspended *S. gordonii* cells were labeled with 20 µl Hexidium Iodide (1.6 mg/ml; Molecular Probes, Eugene, OR) in the dark at 25°C with gentle shaking on a rocker platform for 15–30 min. Unbound dye was removed by washing with PBS and labeled cells were then suspended in 1 ml of pre‐reduced PBS. Cells were diluted to O.D._600nm_ of 0.8 and 1 ml of the labeled cells was added to each well of a 12‐well microtiter plate (Greiner Bio‐one) containing a circular glass coverslip. Plates were incubated for 24 hr on a rotary shaker under anaerobic conditions. The following day, *P. gingivalis* cells were harvested from 10 ml of overnight culture, suspended in 1 ml pre‐reduced PBS containing 10 µl of carboxyfluorescein (1.6 mg/ml; Molecular Probes) and incubated for 30 min at 25°C on a rocking platform. Unbound dye was removed by centrifugation at 3,000 *g* for 2 min. After removing unbound *S. gordonii* from the plates by aspiration, 1 ml of labeled *P. gingivalis* suspension (O.D._600 nm_ of 0.4) was added to each well and incubated for 24 hr on a rotary shaker under anaerobic conditions. To determine the functional activity of the truncated Mfa1 polypeptides, *S. gordonii* cells were pretreated with 1uM of peptide (in PBS) at 25°C for 30 min prior to adding the labeled *P. gingivalis*.

To visualize *P. gingivalis/S. gordonii* adherence and biofilm formation, unbound *P. gingivalis* cells were removed by aspiration and coverslips were washed once with PBS. Biofilms were fixed by incubating the coverslips with 1 ml of 4% paraformaldehyde for 5 min followed by two washes with PBS. The coverslips were then removed, placed face down on a glass microscope slide containing a drop of anti‐fade reagent (Life Technology) and sealed with nail polish. Visualization of biofilms was carried out by laser scanning confocal microscopy on a Leica SP8 confocal microscope (Leica Microsystems Inc.) using a 488 nm laser to detect labeled *P. gingivalis* and a 552 nm laser to detect *S. gordonii*. *Z*‐plane scans of 25 µm in depth were collected at three randomly chosen frames on each coverslip using a *z*‐step thickness of 0.7 µm. Background noise was minimized using software provided with the Leica SP8 and three‐dimensional constructions of the *Z*‐plane scans and quantification of total green and red fluorescence was conducted using Volocity 6.3 Image analysis software (Perkin Elmer, Akron, Ohio). Data were expressed as the ratio of total green (*P. gingivalis*) to red (*S. gordonii*) fluorescence. Each experiment was carried out in triplicate and three independent experiments were conducted for each peptide. A pairwise, nonparametric analysis of variance was used to determine the statistical difference of *P. gingivalis* association with *S. gordonii* between the experimental and control samples. A *p* value of <.05 was considered significant.

### 2.5 SiteMap prediction of a putative binding cleft in Mfa1

The Mfa1 protein structure (Protein Data Bank entry 5NF3) (Hall et al., 2018) was examined using SiteMap v4.5.011 (Schrodinger Release 2017‐4). Standard parameters were used with a maximum of 10 binding sites reported. The top six sites with a sitescore greater than 0.8 were examined further. Residues within 5 angstroms of the potential sites were identified and the highest scoring site (sitescore 1.04) was used for validation by mutagenesis. The five peptidomimetic compounds (pcp‐iii‐201, pcp‐iii‐206, pcp‐iii‐212, pcp‐iii‐293 and pcp‐iv‐20) [Tan, Patil, Luzzio, & Demuth, 2018] were built and minimized using Macromodel (Schrodinger Release 2017‐4) and docked using Glide (Schrodinger Release 2017‐4) in SP mode centered on the highest scoring site from SiteMap.

### 2.6 Site‐directed mutagenesis

Site‐specific mutation reactions were carried out using the Quick‐change II XL Site‐Directed Mutagenesis Kit (Agilent Tech) according to the manufacturer's protocol. Mutagenic primers were designed such that the desired mutation was flanked by 10–15 bp of *mfa1* sequence and are listed in Table 2. The reaction mixture contained 5 µl of reaction buffer, 10 ng of plasmid pTCOW‐Mfa1 template DNA (Park et al., 2005), 125 ng of each primer, 1 µl dNTP mix, 1 µl of *Pfu* Ultra HF DNA polymerase (2.5 U/µl) and water to a final volume of 50 µl. Cycling parameters were as follows: initial denaturation was carried out at 95°C for 30 s, followed by 16 cycles of denaturation at 95°C for 30 s, annealing at 55°C for 1 min and extension at 68°C for 14 min. After amplification, products were immediately transferred to ice. A reaction containing pWhitescript 4.5‐kb and primers supplied by the manufacturer was carried out simultaneously and served as positive reaction control. To eliminate parental plasmid following amplification, PCR products were digested with *Dpn*I for 1 hr at 37°C. Subsequently 1 µl of the digested product was transformed into chemically competent *E. coli* XL10 blue cells (Agilent Tech) by heat shock at 42°C for 45 s. The transformation reaction was transferred into 500 ul of pre‐warmed SOC medium (2% tryptone, 0.5% yeast extract, 10 mM NaCl, 2.5 mM KCl, 10 mM MgCl_2_, 10 mM MgSO_4_ and 20 mM glucose) and incubated at 37°C for 1 hr with shaking at 225 rpm. Positive transformation was selected by plating on LB agar plates supplemented with 100 µg/ml of ampicillin. Five positive colonies were picked for plasmid extraction and successful mutation was confirmed by DNA sequencing.

### 2.7 Random mutation

Random mutagenesis was performed using GeneMorph II Random Mutagenesis kit (Agilent Tech.) to generate additional mutations V238A, I252F and ΔK253. Briefly, reactions contained 2 µg of plasmid pG‐N279, 5 µl of 10X Mutazyme II reaction buffer, 1 µl of 10 nM dNTP, 0.5 µl of primer mix (250 ng each), 1 µl Mutazyme II DNA Polymerase (2.5 U/µl) and 41.5 µl of deionized water. The PCR profile was as follows: denaturation at 95°C for 2 min, 20 cycles comprised of denaturation at 95°C for 30 s, annealing at 60°C for 30 s and extension at 72°C for 1.5 min with final extension at 72°C for 10 min. Following the amplification, PCR products were excised from an agarose gel after electrophoresis, cloned into pGEX6p1 and transformed into chemically competent *E. coli* BL21. Colonies were randomly chosen and plasmids were purified. Successful mutation was confirmed by DNA sequencing.

### 2.8 Complementation of *P. gingivalis* SMF1 with mutated Mfa1

Complementation of the Mfa1‐deficient *P. gingivalis* strain SMF1 was carried out using a modification of the protocol previously described (Park et al., 2005). *P. gingivalis* SMF1 was grown on a blood agar plate under anaerobic conditions for 2–3 days and donor *E. coli* S17‐1 was grown aerobically on a LB agar plate. pTCOW‐mfa1 containing the desired mutation was electroporated into *E. coli* strain S17‐1 and subsequently conjugated with *P. gingivalis* SMF1 using the agar plate method. Briefly, cells from both donor and recipient were scraped from the agar plates and spread on a 4 cm^2^ area on a blood agar plate containing no antibiotics. After incubation for 24 hr, mixed cells from blood agar plates were collected and incubated in TSBY supplemented with hemin (1 µl/ml) and menadione (5 µl/ml) for 1 hr at 37°C under anaerobic conditions. Subsequently, 0.1 ml of the cell suspension was plated on blood agar containing 20 µg of erythromycin per ml and 200 µg of gentamicin per ml and was incubated anaerobically at 37°C for 10 days. Transconjugants were subsequently grown in the presence of antibiotics and the purified plasmid was confirmed to possess the desired mutation by DNA sequencing.

### 2.9 Cell surface expression of mutated Mfa1 polypeptides

Cell surface expression of Mfa1 by the transconjugants was determined using an enzyme‐linked immunosorbent assay (ELISA) after adsorption of *P. gingivalis* strains onto Maxisorp plates (Nunc). Briefly, *P. gingivalis* cells were centrifuged at 3,000 *g* for 5 min and cell pellets were washed there times with PBS. Subsequently, 10^7^ cells were incubated in each well for 2 hr at 4°C followed by washing with PBS to remove unbound bacteria. Bound cells were incubated with rabbit rMfa monoclonal antibodies (1:5,000 dilution) (Covance, Denver, PA) for 1 hr at 37°C. After washing with PBS, wells were reacted with horseradish peroxidase‐conjugated goat anti‐rabbit antibody (1:3,000 [ImmuneReagents Inc.]) for 1 hr at 25°C. Antigen–antibody binding was determined by adding 200 µl of 1× TMB ELISA substrate solution [3, 3′, 5, 5′‐tetramethylbenzidine] (Invitrogen) and the reaction was incubated for 15 min at 25°C. Reactions were stopped using 50 ul of stop solution (0.16 M H_2_SO_4_) and the endpoint was measured at 450 nm in a Victor Multilabel counter (Perkin Elmer).

### 2.10 Mfa1 polymerization

Whole cell lysates were prepared using a modification of the procedure previously described by Hasegawa (Hasegawa et al., 2016). Briefly, *P. gingivalis* strains were grown until early stationary phase in TSBY media supplemented with hemin (5 µg/ml) and menadione (1 µg/ml). Following centrifugation at 6,000 *g* for 5 min, cell pellets were collected and suspended in 1× NuPAGE LDS sample buffer (Thermo Fisher) at the final OD of 2. The cell suspensions were then denatured by incubation either at 60°C or 100°C for 10 min. Following the heat treatment, the whole cell lysate was centrifuged at 20,000 *g* for 10 min to remove cellular debris, electrophoresed in 12% Bis‐Tris Plus gel (Thermo Fisher) and Mfa1 was visualized by ELISA as described above.

## 3 RESULTS

### 3.1 Localization of Mfa1 functional domains

**Figure 1:**
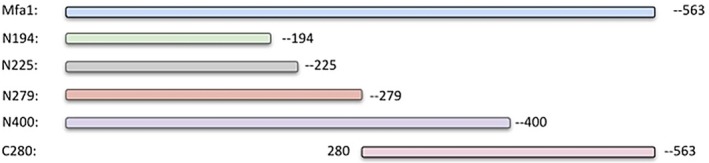
Schematic representation of the series of Mfa1 peptide fragments. The full‐length Mfa1 lacking the signal peptide (21–563aa residues), and N‐terminal peptide fragments N194, N225, N279 and N400 encoding residues 21–194, 21–225, 21–279 and 21–400, respectively, are shown. The C‐terminal peptide fragment, C280, is comprised of Mfa1 residues 280–563

**Figure 2:**
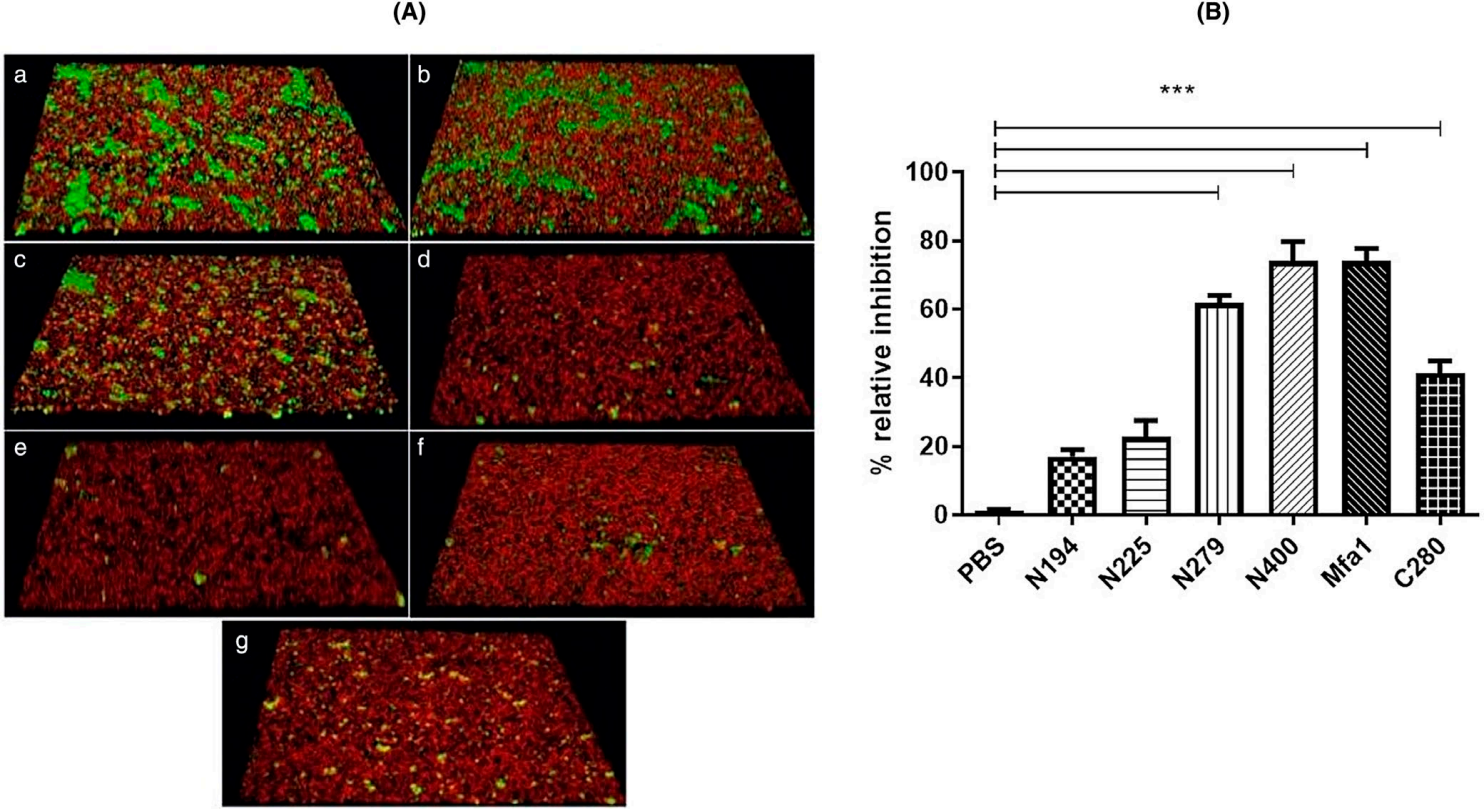
(A) Peptide mediated inhibition of *P. gingivalis* adherence in a dual species biofilm model comprising *S. gordonii* (red) and *P. gingivalis* (green). Panels (a) PBS treated, (b), (c), (d), (e), (f) and (g) were treated with peptides N194, N225, N279, N400, full‐length Mfa1 and C280, respectively. (B) Quantification of relative adherence of *P. gingivalis* and *S. gordonii* was determined by VOLOCITY software. Comparisons of biofilms formed in PBS (control) with peptide‐treated biofilms were carried out using an unpaired *T* test. *p < .05

To identify regions of Mfa1 that contribute to the interaction with antigen I/II, a series of N‐ and C‐terminal truncated Mfa1/GST fusion proteins were constructed and expressed. The truncated Mfa1 polypeptides were purified by removing the GST tag by in column cleavage and were designated as N194, N225, N279, N400 and C280 as shown in Figure 1. The functional activity of these peptides was determined by assessing their ability to inhibit *P. gingivalis/S. gordonii* adherence and biofilm formation using the dual species biofilm model described previously by Patil (Patil et al., 2016). Representative images of biofilms formed in the presence of each peptide are shown in Figure 2a and inhibition results are summarized in Figure 2b. Peptides N194 and N225 were relatively poor inhibitors of *P. gingivalis* adherence (~20% inhibition) compared to the full‐length Mfa1 protein (80% inhibition). In contrast, peptide N279 exhibited 70% inhibition and adherence inhibition by peptide N400 was similar to that of the full‐length Mfa1 protein. Peptide C280 exhibited reduced activity (~40% inhibition) compared to peptides N279 and N400 but was significantly more active than peptides N194 and N225. Together, these results suggest that essential functional residues that contribute to *P. gingivalis* adherence to streptococci reside in the region of Mfa1 comprising amino acids 225–400.

### 3.2 In silico prediction of a putative Ag I/II binding cleft in Mfa1

**Figure 3:**
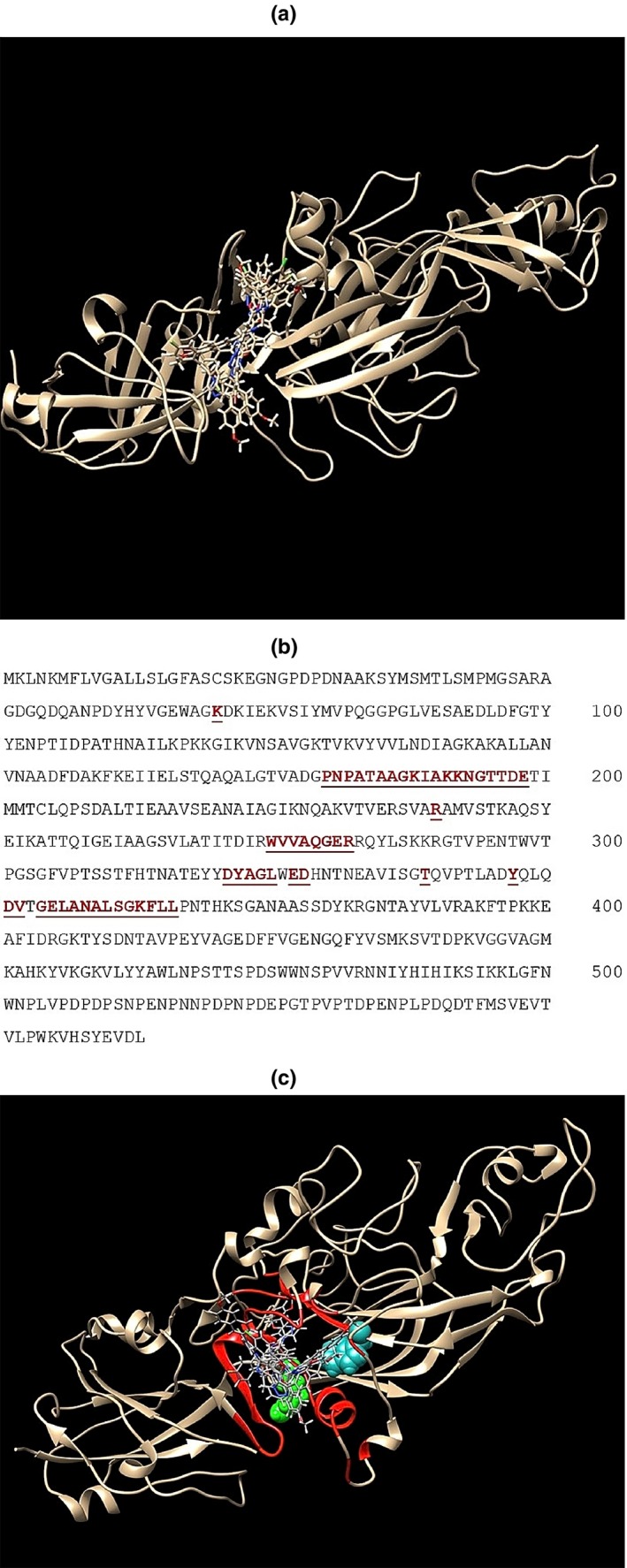
(a) Three‐dimensional structure of the Mfa1 with a composite of five peptidomimetic adherence inhibitory compounds docked in a putative binding cleft. The residues that comprise the predicted binding cleft shown in “a” are shown in red underlined text in the Mfa1 sequence (b) or highlighted in red in the Mfa1 structure (c). The positions of residues R240 and W275 (see text) are shown in green and cyan, respectively

To further highlight the functional region(s) of Mfa1, we took advantage of the recently published three‐dimensional structure of Mfa1 (Hall et al., 2018) to predict putative binding clefts using SiteMap. In addition, a series of in silico docking experiments were conducted using five peptidomimetic compounds that mimic the BAR peptide and were previously shown by Patil et al. (Patil et al., 2015, 2016) to be potent competitive inhibitors of *P. gingivalis/S. gordonii* adherence. As shown in Figure 3a, all five of the mimetic compounds could be docked in the putative binding cleft that exhibited the highest sitescore by SiteMap. Amino acids of the Mfa1 protein that comprise the putative binding cleft are highlighted in red and underlined in Figure 3b.

**Figure 4:**
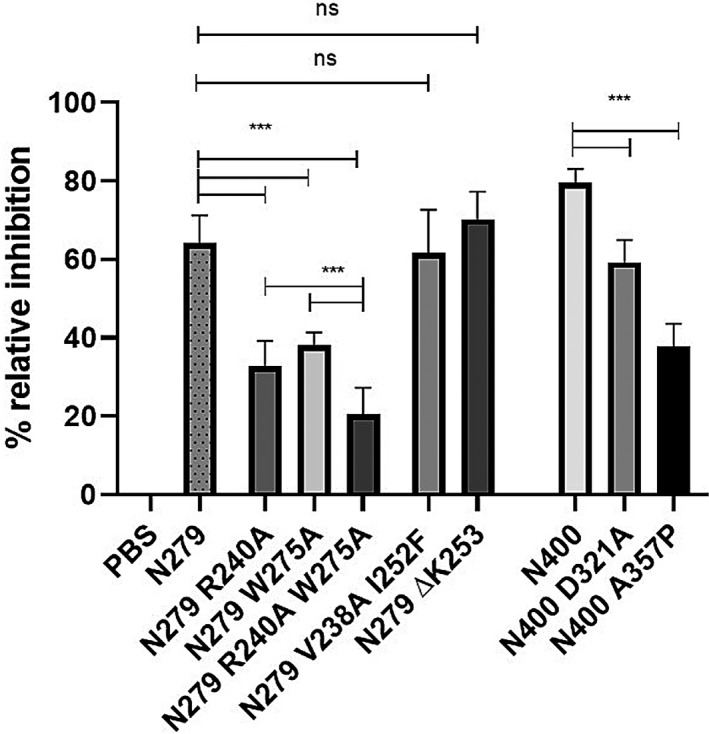
Inhibition of *P. gingivalis* adherence to *S. gordonii* by mutated Mfa1 peptides. Biofilms treated with parent and mutated peptides were compared and analyzed using an unpaired *T* test. ***p < .05, ns, not statistically significant

To validate the predicted binding cleft, a series of Mfa1 site‐specific mutant peptides that targeted residues and putative motifs predicted in Figure 3b to comprise the binding cleft were constructed and expressed. Since the results of the truncated Mfa1 peptides in Figure 2b indicated that the region comprising residues 226–279 was important for *P. gingivalis* adherence, site‐specific mutations R240A and W275A were introduced into peptide N279 since both of these residues are predicted by SiteMap to be part of the binding cleft. Additional mutations, D321A and A357P, were also constructed in peptide N400 to disrupt two predicted amphipathic helices in the putative binding cleft (residues 321–329 and 351–364). Finally, several additional residues in peptide N279 that were not predicted to comprise the binding cleft were tested (e.g., V238A, I252F and ΔK253). As shown in Figure 4, peptides N279 and N400 inhibited *P. gingivalis* adherence to streptococci by 66% and 79%, respectively, consistent with the results shown in Figure 2b. Polypeptide N279 containing the R240A or N275A mutations were significantly less potent inhibitors relative to the parent N279 peptide, exhibiting only 32% and 38% inhibition of *P. gingivalis* adherence, respectively. In addition, peptide N279 containing both mutations, R240A and W275A exhibited significantly lower inhibitory activity than either of the peptide fragments containing a single mutation. In contrast, peptide N279 containing the mutations V238A, I252F or ΔK253 showed no significant reductions in inhibitory activity. Furthermore, mutations D321A and A357P, intended to disrupt the two putative helices, also reduced inhibitory activity relative to the parent N400 peptide (79% to 59% and 79% to 38%, respectively). Together, these results provide preliminary validation of the binding cleft predicted by SiteMap and identify specific Mfa1 residues that contribute to adherence of *P. gingivalis* to streptococci.

### 3.3 Complementation of Mfa1‐deficient *P. gingivalis* with site‐specific Mfa1 mutants

**Figure 5:**
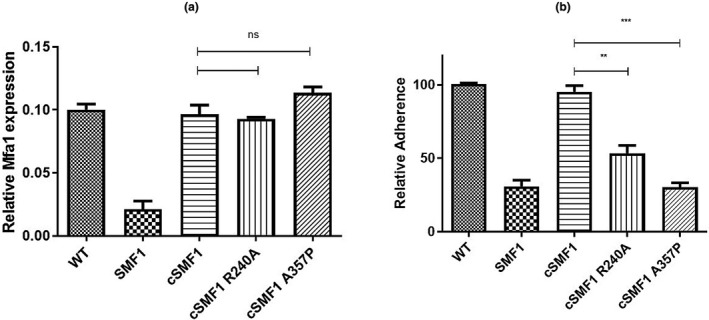
Complementation of *P. gingivalis* SMF1 with wild‐type and site‐specific mutants of Mfa1. (a) Cell surface expression of Mfa1 was determined by ELISA using polyclonal anti‐Mfa1 antibodies. Cell surface expression was normalized to the level of Mfa1 expression in wild‐type *P. gingivalis* 33277. (b) Adherence of *P. gingivalis* to streptococci was determined using a two species biofilm model as described in Materials and Methods. Adherence data were normalized to the level of adherence of the wild‐type *P. gingivalis* 33277 and data were analyzed using an unpaired *T* test. ***p < .001, **p < .05, ns, not statistically significant

To further confirm the functional roles for R240 and A357, full‐length Mfa1 polypeptides containing the R240A and A357P mutations were constructed and introduced into the Mfa1‐deficient strain *P. gingivalis* SMF1. As shown in Figure 5a, cell surface expression of Mfa1 was significantly reduced in *P. gingivalis* SMF1 compared to the wild‐type strain, *P. gingivalis* 33277. Complementation of *P. gingivalis* SMF1 with wild‐type *mfa1* or with the site‐specific mutants restored cell surface expression of Mfa1 to wild‐type levels (Figure 5a). Consistent with its level of cell surface expression, adherence of *P. gingivalis* SMF1 to streptococci was significantly reduced relative to the parent strain 33277 but was restored to wild‐type levels by complementation with full‐length *mfa1*, as shown in Figure 5b. In contrast, although complementation with Mfa1 containing either the R240A or A357P mutations restored cell surface expression, both of these complemented strains showed significantly reduced levels of adherence to streptococci. Indeed, adherence by the complemented strain expressing Mfa1‐A357P was indistinguishable from *P. gingivalis* SMF1 (Figure 5b).

### 3.4 Polymerization of wild‐type and mutant Mfa1 proteins

**Figure 6:**
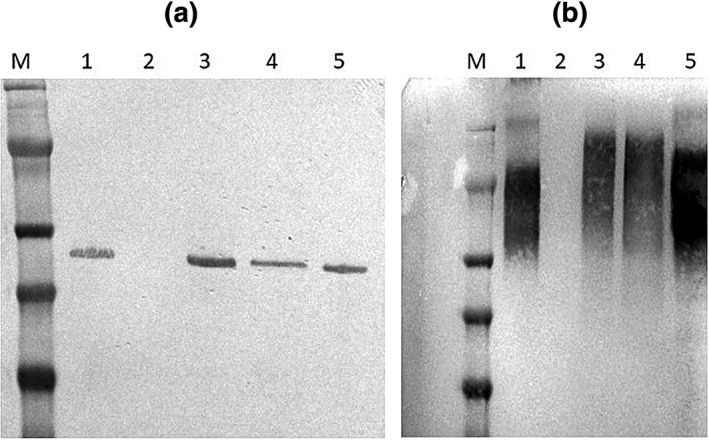
Denaturation of *P. gingivalis* minor fimbriae. *P. gingivalis* cells were suspended in 1× LDS buffer and incubated either at (a) 100°C or (b) 60°C for 10 min. Extracts were electrophoresed in a 12% Bis‐Tris gel and after transfer, Mfa1 was visualized using polyclonal anti‐Mfa1 antibodies. Lanes 1, P*. gingivalis* ATCC 33277; 2, *P. gingivalis* SMF1; 3, *P. gingivalis* cSMF1; 4, *P. gingivalis* cMF1‐R240A; and 5, *P. gingivalis* cSMF1‐A357P; M, size markers

In vivo, *P. gingivalis* Mfa1 is post‐translationally modified by glycosylation (Zeituni, McCaig, Scisci, Thanassi, & Cutler, 2010) and likely polymerizes to form the intact minor fimbrial structure by a donor strand based assembly mechanism involving both N‐ and C‐terminal domains in Mfa1 (Hall et al., 2018; Lee et al., 2018). In addition, intact minor fimbriae resist complete denaturation by SDS unless samples are incubated at 100°C (Hamada, Sojar, Cho, & Genco, 1996; Park et al., 2005). To determine whether the mutations described above affect Mfa1 polymerization, *P. gingivalis* cells were suspended in 1x LDS buffer and incubated at either 100°C or 60°C. As shown in Figure 6a, incubation at 100°C resulted in a single protein band of 67 kDa in all samples except the Mfa1‐deficient SMF1 strain, consistent with the completely denatured Mfa1 polypeptide. In contrast, incubation at 60°C produced a high molecular weight smear in all samples except strain SMF1 (Figure 6b), indicating that incomplete denaturation of the minor fimbriae had occurred. Mfa1 proteins containing either R240A or A357P mutations behaved similarly to the wild‐type protein in the 33277 and complemented cSMF1 strains, suggesting that these mutations do not affect Mfa1 processing or polymerization and that the strains expressing the mutated Mfa1 polypeptides are still capable of producing intact minor fimbriae.

## 4 DISCUSSION

Heterotypic community formation of *P. gingivalis* with oral streptococci is driven by a protein–protein interaction between the minor fimbrial antigen (Mfa1) of *P. gingivalis* and streptococcal surface antigen I/II, for example, SspB of *S. gordonii* (Brooks et al., 1997; Demuth et al., 2001; Park et al., 2005). This interaction has been shown to modulate the virulence potential of *P. gingivalis* (Hajishengallis & Lamont, 2016; Kuboniwa & Lamont, 2010) and may also be important for initial colonization of the oral cavity by *P. gingivalis*. Therefore, disruption of heterotypic community formation by targeting the Mfa1/antigen I/II interaction may represent a potential therapeutic approach to control *P. gingivalis* colonization and virulence (Daep et al., 2011; Sztukowska, Roky, & Demuth, 2019; Tan et al., 2018). The region of antigen I/II involved in the interaction with Mfa1 has been extensively characterized (Daep, James, Lamont, & Demuth, 2006; Daep et al., 2008, 2011; Daep, Novak, Lamont, & Demuth, 2010) and these studies led to the development of both peptide and small molecule peptidomimetics that potently inhibit *P. gingivalis*/streptococcal adherence in vitro and significantly reduced *P. gingivalis* virulence in vivo (Daep et al., 2011; Patil et al., 2016; Patil, Tan, Demuth, & Luzzio, 2019; Tan et al., 2018). However, the interacting interface of Mfa1 that drives this protein–protein interaction has not been well characterized.

Based on peptide mapping, our results indicate that important functional residues and/or motifs of Mfa1 reside between residues 225 and 400 of the protein and truncated peptides comprising this region inhibited *P. gingivalis* adherence to streptococci as effectively as the full‐length Mfa1 protein. In addition, analysis of the three‐dimensional structure of Mfa1 using SiteMap identified a putative ligand binding cleft in which five small molecule mimetics of the BAR peptide could be readily docked. Interestingly, many of the residues within five angstroms of the peptidomimetic ligands are present in the 225–400 residue region of Mfa1. BAR peptide can also associate with this site but docking studies using the more structurally complex peptide will require further refinement. The functional properties of several of these residues were confirmed by site‐specific mutagenesis and peptides containing the R240A, W275A or A357P mutations were significantly less effective inhibitors of *P. gingivalis* adherence that the parent peptide or full‐length Mfa1. Amino acids R240 and W275 form hydrogen bonds with residues that stabilize the binding cleft and those residues are directly accessible in the binding site pocket. The A357P mutation likely disrupts a short amphipathic α‐helix in the binding site pocket and we can speculate that this region may interact with the amphipathic VQDLL motif of BAR peptide which is essential for its interaction with Mfa1.

Consistent with this, complementation of an Mfa1‐deficient strain of *P. gingivalis* with full‐length copies of Mfa1 containing the R240A, W275A or A357P mutations did not restore *P. gingivalis* adherence whereas complementation with native Mfa1 restored the wild‐type phenotype. Even though the Mfa1/Ag I/II interaction is essential for *P. gingivalis* adherence and stable biofilm formation, it should be noted that inactivation of *mfa1* did not completely eliminate adherence to streptococci in vitro. One explanation for this is that the major fimbrial subunit protein, *P. gingivalis* FimA, can also interact with streptococcal cell surface GAPDH (Maeda et al., 2004); however, this interaction by itself is insufficient to promote stable *P. gingivalis*‐streptococcal biofilms (Lamont et al., 2002). Together, these results validated the ligand binding cleft identified by SiteMap, identified specific amino acids that contribute to *P. gingivalis* adherence and suggest that the central region of Mfa1 is essential for the interaction of the minor fimbriae with the BAR motif of streptococcal antigen I/II.

Biogenesis of the minor fimbriae in *P. gingivalis* requires proteolytic and post‐translational processing of Mfa1 and subsequent polymerization to form the fimbrial structure, most likely via a donor strand based exchange mechanism. Polymerization of Mfa1 may involve β‐strands at both the N‐ and C‐terminal regions of Mfa1 (Hall et al., 2018; Lee et al., 2018). In contrast, our results indicate that the central region of Mfa1, between residues 225 and 400, is required for *P. gingivalis* adherence to streptococci and consistent with this, the highest scoring ligand binding cleft identified by SiteMap is mostly comprised of residues in this central domain. Furthermore, the polymerization of mutated Mfa1 peptides that are defective in streptococcal adherence was similar to the wild‐type Mfa1 protein, suggesting that independent domains of Mfa1 are required for fimbrial biogenesis and *P. gingivalis*‐streptococcal adherence. Consistent with this, it was recently shown that peptides CT1 and CT2 which are both derived from the C‐terminal region of Mfa1 encompassing residues 546–563 inhibit Mfa1 polymerization (Alaei, Park, Walker, & Thanassi, 2019). Peptide CT2 also inhibited *P. gingivalis*‐streptococcal biofilm formation and functioned by interfering with minor fimbrial biogenesis. In contrast, the BAR peptide and the BAR peptidomimetics function as competitive inhibitors of streptococcal adherence and have no effect on minor fimbrial biogenesis. The mature minor fimbriae of *P. gingivalis* also contain three additional tip proteins, Mfa3, Mfa4 and Mfa5. These proteins appear to play a role in the assembly of the tip complex itself and its incorporation into the fimbrial shaft and are required for optimal surface expression of the minor fimbriae (Hasegawa et al., 2016, 2013; Ikai et al., 2015). While Mfa1 has been shown to interact with Mfa3 (Lee et al., 2018), there is little information to suggest that the tip proteins contribute directly to *P. gingivalis* adherence to streptococci. Indeed, purified recombinant Mfa1 in the absence of the tip proteins potently inhibits *P. gingivalis* adherence, suggesting that Mfa3, Mfa4 and Mfa5 do not play a major role in the interaction with streptococcal antigen I/II.

Although several specific amino acids and/or structural motifs of Mfa1 were shown to be important for its interaction with Ag I/II, the functional properties of other residues predicted to comprise the ligand binding cleft have yet to be determined. For example, K70 and Mfa1 amino acids 180–194 were identified as putative cleft residues; however, the truncated peptide N225 was only a poor inhibitor of *P. gingivalis* adherence. This suggests that these residues may not interact directly with Ag I/II (or the BAR peptide), or alternatively, that they may also require the contribution of downstream residues. Since Mfa1 has been recently crystallized (Hall et al., 2018), it may be possible to co‐crystallize the protein with the BAR peptide or with recently developed peptidomimetics of BAR (Patil et al., 2019) to generate a more complete picture of the Mfa1‐Ag I/II interacting interface. Ultimately, a thorough understanding of the mechanism of the Mfa1/Ag I/II interaction will facilitate structure‐based drug design and the development of potential therapeutics that may limit *P. gingivalis* colonization of the oral cavity.

## CONFLICT OF INTEREST

The authors declare no conflicts of interest.
